# Book Reviews

**Published:** 1820-01

**Authors:** 


### CRITICAL ANALYSIS

OF

### RECENT PUBLICATIONS, IN THE DIFFERENT BRANCHES OF MEDICINE AND SURGERY.

"I would have men know, that, though I reprehend the easie passing over of the causes of things, "by ascribing them to secret and hidden vertues and properties; (for this hath arrested and laid "asleepe all true enquiry and indications;) yet I doe not understand but that, in the practical "part of knowledge, much will be left to experience and probation, whereunto indication cannot "so fully reach: and this not only in *specie*, but in *individuo*. Yet it was well said, *Vere scire* "*esse per causas scire*."—BACON.

***Surgical Essays.*** By ASTLEY COOPER, F.R.S. Surgeon to Guy's Hospital; and BENJAMIN TRAVERS, F.R.S. Surgeon to St. Thomas's Hospital. Part II. 8vo. pp. 239; with Plates. London: Longman and Co. 1819.

THIS Part is composed of three Essays: 1, *on Dislocations;* 2, *on Unnatural Apertures in the Urethra;* and 3, *on a Species of Encysted Tumor;* the whole by Mr. COOPER.

The *Essay on Dislocations* is given as an addition to the former one on the same subject, and is almost wholly composed of histories of cases collected from various sources, extraneous to Mr. Cooper's own observations: but he has generally made some reflections on them, which, as well as the cases themselves, will be noticed by us, when they present any thing either particularly remarkable or generally interesting: the common-place matter will not, of course, detain our attention. We shall make no preliminary remarks on the value of the contents of this book, but proceed to the examination of it in the manner above indicated. It commences with a paragraph, which we transcribe, because some extraordinary arguments are founded on the observations it contains, in another part of this essay: our remarks on it will be hereafter produced.

"It is a curious circumstance, and one of which I was informed by Mr. Cline, that Mr. Samuel Sharpe, who was surgeon to Guy's Hospital, and had a large share of practice in this metropolis, did not believe that a dislocation of the thigh-bone ever occurred. This want of knowledge in a very excellent surgeon, for the time at which he lived, can only be imputed to the few opportunities which then offered of pursuing morbid anatomy; for he must frequently have seen the accident in the living subject, but never having examined it in the dead, was lead to believe that the appearances of dislocation had arisen from some other cause."

The case first related, is one of dislocation of the femur on the dorsum of the ilium. The history of it is given, apparently, to show the state of the limb when that species of luxation has been suffered to remain unreduced. The attending surgeon, whose name Mr. Cooper has considerately suppressed, attempted to reduce the bone immediately after the accident; "but all his efforts were unavailing," Mr. Cooper observes, "as, unfortunately for the patient, pulleys were not employed." We notice this, in order to show that Mr. Cooper favours the use of pulleys on such occasions; a sort of aid which, we remark with pleasure, is but little resorted to by the generality of English surgeons.

The second is also a communicated case: it is an instance of dislocation of the right thigh, with the head of the bone in the foramen ovale. The patient suffered a severe contusion of the head at the time of the accident; "the symptoms of which," Mr. Cooper says, "precluded, at this time, the possibility of an attempt at reduction." But it appears, that, in fourteen days, the patient was so far recovered as to be able to walk with crutches. Mr. Cooper did not see him until ten months had elapsed: his remarks, after a description of the appearance of the limb, are, "No attempt was made to reduce the limb; the injury to the head might have rendered it dangerous in the commencement; and, at the time I saw him, there was no chance of success." An expression of censure of the conduct of the attending surgeon, (whose name is not mentioned,) would have here been well applied. We do not, indeed, discern why the case should be related, except as a specimen of the results of the neglect of such an injury, and as a basis for some remarks indicative of the practice that should have been employed. The reader will recollect that the patient was limping about at the end of a fortnight from the time of the accident; and that dislocations of the same kind have been reduced after four, five, and even six, weeks.

A case of dislocation of the femur on the dorsum of the ilium, in a child seven years of age, ensues: it was easily reduced by Mr. Daniel, one of Mr. Lucas's dressers, at Guy's Hospital.

The fourth, fifth, and seventh, cases, are instances of "dislocation of the head of the thigh-bone into the ischiatic notch." This accident can only happen when the thigh is bent to an angle with the body, or from great violence acting on the hip itself in a certain manner, and is, on that account, apparently, the most rare species of luxation of the hip-joint, if we except the one downwards and backwards;—a thing spoken of in books as possible, but which no surgeon seems to have witnessed.

Mr. Cooper makes a remark on this subject that is original, we believe, and is certainly of importance in its application to practice. In treatises on Dislocations, the limb is said to be somewhat longer than natural, in this accident; but Mr. Cooper shows that, by looking at the skeleton, we should perceive that it must, in certain positions of the pelvis, be somewhat shorter:

and this was the case in the instances he has here adduced; the difference varied from half an inch to about an inch. Mr. Cooper has not expressly designated the relative position of the body and limb in which the limb is shorter than natural: it was, most probably, on standing upright; since, on lying on a flat surface, especially if the nates are very prominent, it seems apparent that the extremity would be lengthened, or at least as long as the one on the opposite side. This will be understood on turning to the skeleton; where it will be seen that, as the axis of the pelvis varies with respect to the spine, the ischatic notch will be either a little above or below a line drawn transversely through the centre of the acetabulum. Mr. CHARLES BELL, in the last Part of his "Surgical Observations," pointed out some other interesting circumstances respecting the varying axis of the pelvis, that were not hitherto generally regarded with sufficient attention; to which we beg to refer the reader.

We shall transcribe the account given by Mr. Chapman, a dresser at Guy's Hospital, of the manner in which Mr. Cooper reduced the case of the accident above designated, which came under his own observation.

"He ordered so much blood to be taken from the arm as to produce a feeling of faintness. A table was placed in the centre between two staples, upon which the patient was laid on his right side; a girt was passed between the scrotum and the thigh, and carried over the pelvis to the staple behind him; and thus the pelvis was, as far as possible, fixed: a wetted roller was carried around the lower part of the thigh just above the knee, and a leather strap buckled on it, to which the pulleys were fixed, and to a staple before the limb. The body was bent at right angles with the thigh, and it crossed the upper part of the other thigh: then the extension with the pulleys was begun, and gradually increased until it became as great as the patient could bear. An assistant was then directed to get upon the table, and to carry a strong band under the upper part of the thigh; by which he lifted it from the pelvis, so as to give an opportunity for the head of the bone to be turned into its socket. Mr. South, who held the leg, was directed to rotate the limb inwards; and the bone, in thirteen minutes, was heard to snap suddenly and violently into its socket."

Mr. Cooper says, "It is to be remembered, that there is no such accident as a dislocation of the hip downwards and backwards." Perhaps it would be more correct to say, that an accident of this kind may occur, but that the head of the femur would not remain in such a situation; the muscles drawing it either into the ischiatic notch, or just behind the acetabulum.

The other two cases of this species of luxation, occurred to the observation of Mr. ROGERS of Manningtree, and Mr. WICKHAM of Winchester. In both instances, the reduction was readily effected by the surgeons above designated.

Of the eleven cases of dislocation of the hip-joint here related, the head of the femur was, in seven instances, upon the dorsum of the ilium; in three, in the ischiatic notch; and in one, on the foramen ovale.

Mr. Cooper concludes this subject with the following remarks:

"It is really highly gratifying to observe the difference of knowledge in the profession at the present period, when compared with that of fifty years ago. What should we think of a surgeon in the metropolis, in the present day, with all his opportunities of seeing disease in the large hospitals of this town, who doubted the existence of a dislocation of the thigh, when we find our provincial surgeons immediately detect the nature of these injuries, and directly succeed in their attempts to reduce them. Let them never forget, however, that it is to their knowledge of anatomy that they are indebted for this superiority, and more especially to morbid anatomy."

Mr. Cooper seems here to allude to the notion of Mr. Samuel Sharpe,—a notion that cannot much surprise us, because we witness every day opinions almost as strange, in men equally respectable; but the arguments Mr. Cooper has founded on them, present one of the most extraordinary specimens of reasoning perhaps ever witnessed: the *ergos* of Launcelot Gobbo cannot be compared with them. Proceeding in the same manner, we might, by placing the nosological table before us, and adducing, successively, the *bizarre* notions of some physician of the present day respecting every disease it alludes to, and comparing those with the most judicious opinions entertained respecting them a century since, we might in this way easily show that the state of medical knowledge in England, at that period, was far superior to what it is at the present time. Most of our readers have, no doubt, in an undisturbed corner of their library, an old book, covered with dust, called *Wiseman's Chirurgery;* nobody, of course, will read it now-a-days,—it is filled with simple histories of cases and cures only; but, amongst those, is an account of the new discovery that the hip-joint is sometimes dislocated, and of the signs by which the accident may be known. In taking a glance at the worn covering of the writings of the father of English surgery, our eyes were caught by those of Hey, of Park, Gooch, and many others of our *provincial* surgeons, who *volitant vivos per ora virum*, although they have personally ceased to exist; and we feel vexed to find those who have succeeded them placed in such a point of view as they are done in the curious paragraph just transcribed.

Mr. Cooper, with the view of placing fractures of the neck of the femur in comparison with dislocations of the hip-joint, deviates from the express subject of this essay, to adduce some observations on the former of those accidents. With a view to justify, in some degree, the character of one of our most esteemed surgeons, we transcribe the following paragraph: if it may be considered of general application, it will show that his notion on the subject under consideration was not so extraordinary as it might at first appear.

"Such accidents," [fractures of the neck of the femur,] Mr. Cooper observes, "are more frequent than dislocations of the os femoris, which is evinced by the comparative number we admit into our hospitals; being seldom without an example of the fractured neck of the thigh-bone, whilst the cases of dislocation, upon the average, do not exceed one in a year."

On this subject Mr. Cooper has advanced nothing that is novel, either in respect to observations or to useful and well-founded opinions; though, by his account of his consultation with a practitioner who was not aware that fractures of the neck of the femur within the capsule of the joint have never been known to unite, in any well-determined instance, by the ordinary mode of bony union, and holding forth this as his theme, he insinuates that he is about to produce what is generally unknown,—not because such men will not read, but because such knowledge is not elsewhere to be acquired in writings on surgery. Why does Mr. Cooper act thus? Why does he always take the conduct of wilfully-ignorant men, instead of the best conduct and opinions generally prevalent amongst us, as the subject for comparison to his own? A person unacquainted with our literature and general practice would, from Mr. Cooper's writings, be impressed with an idea that English surgeons are at least a century behind those of the rest of Europe in professional knowledge. This is not a mere supposition: it happened in many instances from the perusal of the former part of these Essays, and has been pointed out to our disgrace.

Fracture of the neck of the femur externally to the capsular ligament, dislocations of the knee-joint, fractures and dislocations of the patella, and dislocations of the ankle-joint, are successively considered in the remaining part of this essay: but we are unable to produce from it, either any thing practically useful, that a well-educated surgeon could not refer to in some former author; or precepts for conduct, which one of the most ordinary judgment would not necessarily deduce from his own observations and previous acquirements.

We cannot discern Mr. Cooper's meaning in writing this essay; for, though he has given histories of most of the different species of dislocation of the principal joints, and detailed accounts of the measures employed in their treatment, yet, as he has not risen to general principles, he cannot have intended to write a complete dissertation on this subject: it is therefore not adapted for the elementary instruction of students ; and those who have acquired such instruction elsewhere, will be vexed at having been led to spend the time necessary for the perusal of above two hundred and fifty pages, for so small an addition to their store of useful knowledge. On ordinary occasions men easily avoid this, by throwing aside a book, if they find, after reading a few pages, that they are not likely to obtain from it the information they expected: but here many persons will, probably, be induced to go through the whole, hoping to the last to find those deep reflections, comprehensive views, and principles of general application, that should be formed after long and extensive observation. Why did HIPPOCRATES exclaim, as a prelude to his *Aphorisms*, Ο βιος βραχὺς, ἡ και τέχνη μακρὴ! and in that immortal work comprise the precepts deduced from the experience of seventeen successive generations; but, because he considered that, if every minute particular of each individual variety of disease, with all the accidental circumstances connected with it, and a minute detail of the treatment used in each case, were to constitute the sources of information; no life would be sufficiently long to permit any man to acquire a general knowledge of his art, for ten thousand volumes would hardly comprise the history of diseases; and, consequently, no one could tell how to act in cases of common occurrence, until he had found out a similar instance in the records of his library.

The subject of the second essay will be discerned from the paragraph with which it commences:

"That openings made into the urethra by cutting instruments are healed without difficulty, is evinced by the operation for the stone in the bladder, in which this canal is extensively opened ; and in the removal, by incision, of a calculus lodged in any part of the urethra. But, when apertures are formed in this part, either from diseased states of the constitution and of the urethra, or from disease in the canal only, and when they are accompanied by any considerable loss of substance in the urethra and corpus spongiosum, they are generally very difficult of cure."

An interesting case is related, in which an ulcer of the latter kind communicated with the cavity of the rectum ; for which the following operation was performed on the patient, with a successful result:

"I introduced a catheter into his bladder," says Mr. Cooper, "and my finger into the rectum, and then made an incision, as in the operation for the stone, in the left side of the rhapha, until I felt the staff through the bulb. I then directed a double-edged knife across the perineum, between the prostategland and rectum ; intending thus to divide the fistulous communication between the urethra and the bowels. A piece of lint was introduced into the wound, and a poultice was applied over it. When the lint was removed, the urine was found to take its course through the opening in perineo. The aperture in the rectum gradually healed, and that of the perineum quickly closed; after which the urine took entirely its natural course. Whilst the wound which I had made was healing, one of the testicles became enlarged and inflamed, as I supposed, from the irritation on the extremity of the vas deferens, or in sympathy with an irritated vesicula seminalis on that side. This inflammation left some hardness of the epidydimis, but no further inconvenience; and the urine has never since deviated from its natural channel."

The history of another case is given, where the fistulous ulcer was situate anteriorly to the scrotum, and for which the ordinary measures had been employed without success.

"Feeling it would be quite useless to repeat these trials," Mr. Cooper observes, "and seeing that the scrotum formed two-thirds of the opening, and the skin of the penis the other one-third, I thought that it might be possible to heal it upon the principle of the contraction of the skin in cicatrization. In June 1818, I applied the nitrous acid upon the edge of the fistulous orifice and upon the skin, to the extent of three-quarters of an inch around it: the skin sloughed superficially, formed granulations, and healed. It soon afterwards began to contract, so as to show that the principle would ultimately much diminish the orifice. In the month of October succeeding, I again applied the acid, and with increased effect. At the end of November the application was repeated by himself; and the opening, from the size of a pea, was reduced to that of a pin. On January the 22d, 1819, it was again touched, but very lightly. In March the caustic was last applied; and in a fortnight the orifice was closed, and not the smallest quantity of urine has since passed."

The most important case is the ensuing one, in the treatment of which the Tagliacozzian art was resorted to, and with consequences that equalled the most favourable expectations. This is a gratifying result of Mr. Cooper's efforts to improve the practice of his art. We shall give an abstract of the history of the disease, and of the operation.

In the beginning of July, 1818, Mr. H—, aged fifty-six, suffered a severe attack of inflammation of the penis and scrotum, attended with considerable swelling of the parts; apparently arising from a neglected stricture of the urethra. It was treated by purgative medicines, fomentations, and poultices. On the 9th of July a large abscess was opened, opposite to the bulb of the urethra, which discharged a great quantity of very fetid matter. On the 30th of July, Mr. Cooper introduced a silver catheter into the bladder; with difficulty, however, on account of the resistance of two firm strictures. The catheter was worn for three weeks; during which time another abscess burst, at the under part of the urethra, immediately behind the scrotum. The swelling and inflammation gradually abated, and the fistulous orifice behind the scrotum closed; but that before it continued to extend, until it was sufficiently large to admit with ease a full-sized catheter. In this state it continued nearly four months, not showing the slightest disposition to heal. Nitrate of silver, and other stimulating applications, had been employed. Attempts were made to produce union by adhesion, but without success, it being impossible to keep the edges in contact. Mr. Cooper then resorted to the following operation:

"*December 9th.*—An elastic catheter being passed into the bladder, the callous edges of the opening were pared off, so as to produce an entire new surface: a portion of integument was then dissected from the scrotum, (leaving it attached at the upper part,) and turned over upon the wound, to which it was exactly fitted: this was held down by four sutures, covered by small strips of adhesive plaster. A bandage was applied to support the scrotum, and the patient placed on his back in bed."

On the following day a little matter was observed to pass by the side of the catheter, at the extremity of the penis; some urine escaped by the wound, and there was much aching pain about the part. The scrotum then became swoln and inflamed, and a small quantity of urine continued to flow through the wound during four days longer. At the end of this time the edges of the flap appeared in perfect apposition with the parts beneath; but the skin was thick and œdematous. The sutures all retained their hold. Three days afterwards, a little urine again escaped by the wound. The catheter having become stopped up, it was withdrawn, and another introduced. Dec. 20th. Much acrid discharge from the wound, (principally from a small opening on the right side;) considerable excoriation of the cuticle. Dec. 21st. The upper and left side of the flap appears perfectly united. Dec. 23d. The whole of the sutures were removed, as they kept up considerable irritation; the discharge from the wound passes only by the small sinus opening on the right side. Dec. 27. The catheter again withdrawn, and another introduced, which passed with but very little difficulty; several hairs sprouting out on the flap of skin; the discharge still continuing from the sinus, the effect of pressure was tried in preventing it. Dec. 2[illegible]th. The compress has completely stopped the discharge by the sinus. January 5th. A quantity of urine which passed through the urethra by the side of the catheter, produced no effect upon the wound. Jan. 10th. The patient sat up without the catheter between three and four hours; it was afterwards withdrawn daily, but he was not allowed to pass his urine without it. Jan. 15th to 31st. The catheter was withdrawn for a few hours every day; slight discharge from the wound. Feb. 1st. Had an evacuation from the bowels during the time the instrument was out of the bladder, attended with a considerable discharge of urine by the natural passage; no urine passed by the wound.

"*February 2nd to March 2nd.*—As the flap of integument and adjacent parts had still rather an irritable appearance, and an occasional oozing was observed from a very small orifice on the right side of the wound, it was thought improper to hazard a repetition of this experiment. The catheter was therefore introduced twice a-day, and continued in the bladder at night.

"*March 3d.*—He now began to pass his urine without the aid of an instrument, using it only once a-day, to prevent the return of an old stricture, of very long standing, in the membranous part of the urethra: after the first effort it came in a tolerably free stream, more so than it has done for many years. A weak solution of sulphate of zinc soon removed the irritable appearance of the integuments.

"*May 8th.*—A common bougie was substituted for the elastic catheter, and introduced once every day till the latter end of September; since which time he has passed it but once in two days. He is now, October 14th, in perfect health. The stream of water becomes gradually fuller and stronger; the penis is somewhat drawn down by the contraction of the integument; and the small pouch which was formed by the ligatures at the upper part of the flap, is removed."

After so long a period, the fear that the unnatural stimulus of the urine to the skin would produce ulceration, which, on hearing of this operation, we had entertained, may apparently be considered invalid; but yet we think that some time must still elapse before the permanency of the cure may be fully depended on.

The species of *encysted tumor* to which Mr. Cooper here confines his observations, is that

—"which is situated just under the skin, and is so frequently seen upon the head, the face, and upon the back; and occasionally, but less frequently, under the skin upon other parts of the body.

"Having been myself the subject of one of these tumors, upon my back, I was led to observe it with more than common attention; and am induced to hope that I shall be able to show from what source these swellings derive their origin.

"The encysted tumor is generally nearly globular, and, when seated on the head, feels very firm, but upon the face it possesses a fluctuation more or less obscure. The skin over it is generally uninflamed; but it is now-and-then streaked with blood-vessels, which are larger than those of the surrounding parts.

"In the centre of the tumor on the skin, it often happens that, in this early state, a black or dark-coloured spot may be seen, which sometimes continues through the whole course of the disease; but this is by no means uniformly the case. In general, they are unattended with pain; are never in themselves dangerous; and require removal, from the parts in which they occur, and the defect in appearance they produce. They move readily within the cellular membrane, if they are free from inflammation; but the skin in general does not easily move over them."

Mr. Cooper thinks these tumors are in some degree hereditary; perhaps his meaning will be evident in his own words. The facility with which he forms conclusions will, at least, be discerned in the following paragraph:

"They are in some degree hereditary; for often I hear a patient observe, I have several swellings upon my head, and my father (or my mother) had several.

"They also occur in several of the same family. I was asked by Dr. Pacifico to remove some of these tumors from the head of one of a family who resided near him in Bury-street; and, when I had accomplished this, another said, And I will be obliged to you to do the same; and then a third made the same request."

These swellings, Mr. Cooper observes, are found to adhere to the skin, but by the cellular membrane only, on the greater part of their surface; within the cyst there is a lining of cuticle which adheres to its interior; and several desquamations of the same substance are formed within the first lining, apparently secreted at various periods of the growth of the cyst.

"The substance which is contained within the bag," he continues, "has the character, to the eye, of coagulated albumen; but, as it varies much, this swelling was formerly absurdly named, according to the appearance of its contents, atheroma or meliceris,—a name which only expressed different states of the substance contained in the same disease.

"If the vessels which nourish these cysts are injected, they are found to be but of small size, although they are numerous.

"These cysts sometimes contain hair, when they are situated upon the temple and near the eye-brows, and in other hairy parts of the body. The hairs have no bulb or canal, and differ therefore from those which are produced in those surfaces of the body which naturally form hair."*

Sir Everard Home, in the Philosophical Transactions for the year 1791, Mr. Cooper also remarks, clearly showed that the horny excrescences sometimes met with in human beings, owe their origin to these cysts: such is the opinion, also, of Prof. Meckell.

Mr. Cooper's notion respecting the nature of this tumor seems, from the foregoing and the following arguments, to be correct. He says,

"With respect to their origin, I believe, this tumor arises from a follicle extremely enlarged, and incapable of discharging its contents from an obstruction of the orifice, by which it opens on the surface of the skin.

* These cysts, in the sheep, sometimes contain wool.

"A follicle is a glandular pore, which are found in numbers on the surface of the skin, more especially about the face and head.

"These follicles appear, upon superficial examination, to be only pores in the skin; but, upon the introduction of a fine probe, they are found to proceed through the skin into the cellular membrane beneath it. They are productions from the skin, and are lined naturally by cuticle; and their internal surfaces secrete a sebaceous matter, which lubricates and defends the surface of the skin upon which they are found. This matter may be pressed from the follicles of the nose in the form of worms very considerably longer than the skin is deep, and therefore proving that these pores extend beyond the skin."

On one of those follicles becoming obstructed at its termination upon the skin, and the secretion from its internal surface still proceeding, it becomes distended in the manner previously described. The diseased action, too, arising from this or from some other causes, may be rationally supposed to be capable of producing the variety in the character of their contents.

With respect to the treatment of these tumors, Mr. Cooper advises that, "if the follicle can be seen only as a black spot filled by hardened sebaceous matter, a probe may be passed through it, and the sebaceous matter squeezed from the tumor;" or, if violence be requisite to effect this, an incision into them may be made in the same situation. When they have attained a considerable size, or are otherwise productive of great inconvenience, they may be dissected out, by first making an incision into them, and then, by pressing the sides of the skin together, the cysts may be easily everted and removed.

On making a summary of such of the contents of the Essays here presented to the public by Mr. Cooper as bear the character of novelty, we find them to consist, in a remark on the position of the limb in dislocation of the femur on the ischiatic notch; in the history of a case in which the Tagliacozzian art has been successfully applied, to remedy a fistulous ulcer in the side of the urethra; and in the notion that atheroma, melicera, and analogous tumors, have their origin in a morbid enlargement of the subcutaneous sebaceous glands. Of important matter, besides the points just enumerated, they comprise some more or less interesting cases of dislocations of the principal joints; none of which, however, are so in any great degree, excepting a few instances of compound luxations of the ankle-joint: and the chief interest of these consists in their showing that such cases will terminate in a favourable manner, under active and judicious treatment, more frequently than seems to be generally believed. But, it should be remarked, by far the greater proportion of those cases did not occur to Mr. Cooper's observation: he has merely collected and edited them. How far this agrees with the statements made in the preface, is a question on which different persons may, perhaps, form different conclusions; but we, very patiently, wait in expectation of their being realized.

---

*Medico-Chirurgical Transactions.* Vol. X. Part I. London, 1819. Longman and Co.

THIS volume commences with an *Account of the Rheumatic Inflammation of the Eye, with Observations on the Treatment of the Disease*, by James Wardrop, Esq. f.r.s. Ed. Mr. Wardrop has here given the description of a form of disease which serves as an illustration of the principle adopted, apparently, from Bichat and Pinel, which he advanced in his Essays on the Morbid Anatomy of the Eye, that—the inflammation of each particular texture of that organ is accompanied with a distinct class of symptoms, each assemblage characterizing one species of ophthalmia. The author's observations are here, however, not exempt from vagueness, not to say absolute error; for, he says,

"But there are other kinds of inflammation, which derive their character, not from the peculiarity of the texture inflamed, but from being produced from some *specific virus.* Hence the gonorrheal, the syphilitic, the *scrofulous*, the *gouty*, and the *rheumatic*, inflammations of the eye;—all of which are accompanied with symptoms different from those of simple inflammation of any of the textures which compose that organ."

Such a commencement is by no means calculated to prejudice us in favour of the contents of this paper; and, were it not that a similar form of disease has been described by other writers, and is indeed known to all those who have seen much of diseases of the eye, we should have probably passed it over in silence; for we could not hope to furnish our readers with any sound original pathological knowledge, from an author who prefaced them by such remarks as are contained in the paragraph just transcribed.

The connexion between certain forms of inflammation of the eye and what are called rheumatic affections of various parts of the body, was long since pointed out by Dr. Robert Jackson, in his Treatise on Febrile Diseases, by Professor Beer and Dr. Demours, and some of the most interesting cases of this kind on record were given in the thirty-sixth volume of this Journal; but Dr. Warren,* who related them, though he termed them cases of rheumatic inflammation of the eye, did not enter into any precise pathological reflections respecting them. Mr.

---

* The Professor of Anatomy and Surgery at Havard University, in the United States.

Wardrop has indulged in generalities, without adducing any particular observations. He commences his description with a train of words that have no precise meaning: he says,

"The rheumatic opthalmia derives its character both by the peculiarity of the local and constitutional symptoms."

We suppose he intends to signify, what no one need be informed, that the peculiarity of the symptoms *constitute* the character of the disease.

"The red colour which the albuginea acquires in this inflammation," Mr. Wardrop observes, "is not the bright crimson which accompanies the inflamed cornea; nor is the redness confined to one part, as in the pustular ophthalmia. Neither have the vessels that peculiar mode of ramification, nor are the trunks so superficial, nor is there the puriform discharge which accompanies the inflamed conjunctiva. The albuginea acquires a brick-red tinge, or an admixture of yellow with crimson red; and this peculiarity of colour is probably produced by the serous part of the blood being tinged with bile;—an effect likely to take place from the marked derangement of the biliary organs which usually accompanies this disease. The shade of yellow, however, varies a good deal; being in some cases very remarkable, and in others much less perceptible."

The author should have shown the existence of a similar colour of the skin and of the eye in the healthy state, (for, as far as our own observations have extended, only one eye is commonly affected in the first instance,) in order to prove the existence of bile in the blood in this disease. This we never remarked; and it is not improbable that the yellow colour noticed by the author, depends on a certain state of the blood itself in the vessels, rather than on mixture of bile with it. Sir Isaac Newton observed, that the blood reduced to thin laminæ, assumes a yellow colour. This is a matter of some importance, and it should not have been left doubtful.

"The blood-vessels," the author continues, "are generally equally numerous over the whole white of the eye, passing forwards in nearly straight lines from the posterior part of the eye-ball, and advancing close to the cornea; but neither passing over it nor leaving the pale circle around it, which is so striking when either the choroid coat or iris is inflamed. If the vessels be closely examined, the general redness will be found produced more from numerous small ramifications than a few large trunks."

Injected preparations of the eye will show on what the appearances above noticed depend. Opake specks, and sometimes general cloudiness, at length affect the cornea; and, "if closely examined," the author continues, "there is generally one or more parts where the corneal conjunctiva appears to be abraded; and these abrasions are usually near its circumference, though they may also be occasionally observed on all parts of the corneal surface." The conjunctiva, generally, is but little affected; consequently, but little tumefaction of the eye-lids takes place. Sometimes, however, there is a slightly-elevated ring round the circumference of the cornea.

The following paragraph will show the propriety of the arrangement of this disease, according to the principle of the texture particularly affected:

"The seat, as well as the kind of pain, affords striking characters of this peculiar affection. Generally the chief seat of pain, at the commencement of the disease, is in the head, though it sometimes also affects the eye-ball itself. The pain is usually most severe in the temple of the affected side; but it is often seated in the brow, the cheek-bone, the teeth, or the lower jaw. Sometimes the pain is precisely confined to one half of the head, and sometimes there is a severe pain in the cavity of the nose or in the ear. These pains are more of a dull agonizing kind than acute; and, though in this disease the pain be unceasing, yet it varies much in degree, coming on at times in very severe paroxysms, and recurring with great violence when the head is bent downwards. Sometimes the pain is excited by merely touching the scalp, and the patient is unable to rest his head on the affected side, or even lean it on a pillow.

"The pain in most cases is remittent; the paroxysm coming on at four, six, or eight, o'clock in the evening, continuing during the night, being most severe about midnight, and suffering an abatement towards morning.

"In the eye-ball, the patient generally complains more of a sense of fullness and distention, than of pain; and, though there is a great degree of external redness in this disease, the eye does not seem to suffer from exposure to light, for the eye-lids are kept open without appearing to create uneasiness: whereas, in most other inflammatory affections of this organ, even a very moderate quantity of light cannot be endured.

"This peculiarity in the seat of pain in the rheumatic ophthalmia, may perhaps be satisfactorily explained from the texture of the eye which is affected, and the sympathy which that texture has with similar adjacent structures. This particular species of ophthalmia appears to me to be chiefly seated in the sclerotic coat, which, like the dura-mater and membranes lining the nose, frontal and maxillary sinuses, is of the fibrous class; and it is membranes of this kind which are commonly the seat of rheumatism in other parts of the body. The sympathy between all these parts and the eye is reciprocal; for, when the dura-mater is inflamed, irritability and redness of the eye are its concomitant symptoms."

Nothing, however, has yet been said, which shows the propriety of terming the disease *rheumatic;* and the following account of the constitutional symptoms rather point out a different origin:

"Besides the local symptoms, there is always more or less symptomatic fever accompanying rheumatic ophthalmia, which increases along with the pain towards evening, assuming the form of a severe febrile paroxysm. The pulse becomes frequent, and sometimes hard. The tongue is remarkably white and furred, accompanied with a bitter taste in the mouth, more or less nausea, and the skin is hot and dry. The functions of the primæ viæ are much deranged; the appetite being impaired, and the evacuations always changed in quality."

Sometimes the disease goes off without having produced evident lesion of structure; but, in the more severe cases, the inflammation extends to the conjunctiva, ulceration takes place in the cornea, and the aqueous humour is discharged, when the pain ceases. The latter is a very curious and interesting circumstance, and seems to have pointed out to the author the remedial measure on which he is disposed to place the firmest reliance. In two cases which he witnessed, a quantity of thick puriform fluid has formed in the posterior chamber, and burst through the sclerotic coat.

The author has traced the origin of the disease, in many instances, to a sudden change of temperature; in one instance, to the patient's having kept wet clothes on his head when overheated; and in another, it came on after travelling during the night in a carriage with the head close to an open window: and, he adds,

"It not unfrequently follows the operations for cataract, particularly in patients who have previously had rheumatism in other parts of the body. Rheumatism may frequently be observed to attack a joint or part that has been injured."

This use of the term rheumatism shows to what men will resort, who are led by an inclination to propose causes rather than to trace phenomena: besides, the writer has forgot that he, at the commencement of this paper, considered rheumatism as arising from a *specific virus;* and, not considering this, it is an unwarrantable attempt at generalization, to comprise diseases arising apparently from gastric disorder, from cold applied to the head, and from the injury of the organ attending the operation for cataract, under such a term, because the patients may have had "rheumatism in other parts of the body." This would be of no importance, did it not tend to prevent a proper mode of investigating the nature and origin of the disease; and, if incorrect, favour the use of an improper mode of treatment.

Unless the history of the disease be attended to, this inflammation, the author says, may be confounded with the syphilitic; there is, however, some difference in the appearances: in the rheumatic inflammation, "the proper vessels of the sclerotic coat are enlarged, which is the cause of the redness being generally diffused over the whole albuginea; whereas, in the syphilitic inflammation, it is the anterior ciliary arteries passing along the sclerotica on their way to the iris which are chiefly affected; and thence the pale ring which is always observed between the cornea and enlarged vessels in that disease."

It is long since we met with any thing so vague and contrary to correct reasoning, as the statement in the following paragraph:

"In the same manner, also, as rheumatism and gout are often combined in other organs, so are they found affecting the eye. In some instances the rheumatic affection begins first, and then gout succeeds. But there are cases where the inflammation is purely arthritic."

The pure *arthritic* inflammation of the *eye*, Mr. Wardrop however says, may be distinguished from the rheumatic; "for, like the syphilitic, the gouty inflammation chiefly affects the capsule of the aqueous humour, producing thickening of that membrane, effusion of lymph in the anterior chamber, and adhesions of the edge of the iris with the capsule of the lens; and, whilst these changes are going on, the cornea remains perfectly transparent."

Mr. Wardrop has not favoured us with a description of the *hybrid* disease generated by gout and rheumatism; but this is not much to be regretted; for no pathologist, not disposed to reason on vague terms alone, would think it worthy of his attention. Enough has already been said; for, when a person is so ready with terms from which he cannot possibly himself derive any precise ideas, and then appropriates descriptions to them, we suspect the accuracy of these, and think the author more inclined to paint an imaginary picture, than to copy nature.

With respect to the treatment of the form of disease above alluded to, the author says,

"In the rheumatic inflammation of the eye, I have found the evacuation of the aqueous humour attended with much advantage. In those cases particularly where proper remedies had not been employed at an early period of the disease, where there was much pain in the brow or any other part of the head, where the cornea had become dim and clouded, and where vision was impaired,—in all such cases the good effects of the operation were instantaneous: the pain of the head was removed, and very seldom returned, and the transparency of the cornea was restored. When, therefore, the disease has made much progress, and when the symptoms have not been relieved by other remedies, the evacuation of the aqueous humor is a practice from which the most beneficial effects may be anticipated."

It is our duty here to remind the younger part of our readers of the inordinate partiality men are often disposed to feel for measures of their own invention; and, therefore, that they should be at least reserved in resorting to that advised by Mr. Wardrop in the disease in question. Other practitioners treat this disease with tolerable success, by less hazardous means than the evacuation of the aqueous humour of the eye; which, they may be assured, is not a trifling operation in practice, whatever it may be made appear to them by writings.

Mr. Wardrop recommends vesicatories to the neck, and, if the biliary organs are deranged, such measures as are adapted to restore their healthy state; and, if the disease have arisen from chill, he continues, "the functions of the skin should be restored by the judicious employment of sudorific medicines. A couple of grains of antimonial powder, given singly or combined with opium, every four or six hours, is an excellent remedy; and their effects are particularly useful when a dose is taken a little before the evening paroxysm, as it has frequently the effect of allaying pain and producing sleep."

If antimonial powder be administered with the probability of any important effects, it should be given in at least four times the quantity directed by Mr. Wardrop. The same remark will apply to the purgatives directed: "A couple of grains of calomel combined with a few of rhubarb, two or three times given." But the author's medical measures are not very energetic: he continues to observe,

"Little advantage is derived from local bleeding. In a few cases, where there is a tendency to plethora, a very full and hard pulse, and where relief has not been speedily obtained from the use of the other remedies, it may be necessary to take away some blood, either from the arm or by leeches, from the branches of the frontal veins and arteries. In general, however, patients affected with rheumatic ophthalmia, as with rheumatism in other parts of the body, cannot bear bleeding to a great extent. This remedy should therefore be employed with moderation. Indeed, the little relief afforded by bleeding in this disease may be regarded as one of its diagnostic characters."

This is a new and curious view of diagnosis; but let it be remembered, that, if this be waited for, and the practitioner has mistaken the nature of the case, and applied only a few leeches, instead of having abstracted blood copiously, the disease will probably have produced injurious effects that will be irremediable. Mr. Wardrop has not said how he makes leeches draw blood from "the frontal veins and arteries." We never saw them do more than just pierce the vessels of the skin; which, to be sure, are, in reality, branches of the frontal veins and arteries: but there is here an illusiveness; which, however, we should not have noticed on so trifling a point, were it not that the whole paper abounds with similar matter, evidently spun by the imagination, not copied from actual and correct observance.

Fomentation with decoction of poppy-heads, and, when the first stage of the inflammation has passed away, and the febrile symptoms subsided, the vinous tincture of opium, are the topical measures directed: and, "after the *primæ viæ* have been well evacuated, the tongue may still remain very white, and the pulse quicker than natural. In this state, the greatest benefit is derived from the internal use of the cinchona, singly, or combined with the mineral acids." From the disordered state of the stomach, the author continues, it should be only given in small doses; "generally from *five* to *eight* grains, taken in a little warm water, every two hours, or as often as the stomach can receive it, is sufficient. It sometimes purges the bowels considerably; and, when this effect is produced, I have not found it diminish its specific virtues." There are none of our readers, we hope, who do not know how to appreciate those remarks.

Although some points in the preceding observations,—those, especially, designating the peculiar character of the inflammation of the sclerotica, and the distinctness of the seat of the disease,—may be novel in this country, they are not original with the author. Prof. Beer* had previously described it; and he mentions some points respecting its diagnosis of considerable utility, which show his accurate observation, and which seem to have escaped the notice of Mr. Wardrop. Thus, he remarks, when inflammation is seated in the conjunctiva, the vessels will be seen to accompany any considerable motion given to the eye-lids by the hand, whilst they are but little affected by rolling of the eye itself: on the contrary, when the sclerotica is the seat of the disease, the vessels will be seen moving with the eye, whilst they are not affected by motion of the lids. The reason of this must be obvious to every anatomist. Although Prof. Beer considers this disease as analogous with that affecting the fibrous structure of other organs termed rheumatism, yet he advises the use of copious blood-letting from the arm in almost every instance, joined with leeches to the temples, and other antiphlogistic measures. M. Demours† also speaks of this disease, and has regarded it in a similar manner to that above mentioned. In cases of the more chronic kind, especially in those where the symptoms have an intermittent character, he also advises the use of bark; but, when he has employed it, it has been in much larger doses than those directed by Mr. Wardrop.

With regard to the use of mercury, it is generally agreed, that it is but rarely apparently beneficial, and is often evidently injurious.

---

* In his *Lehre von den Augenkrankheiten, als Leitfaden zu seinen öffentlichen Vorlesungen entworfen.* Wien, 1813.

† *Traite des Maladies des Yeux,* &c. Paris, 1818.

***Memoir on a new Mode of treating Bronchocele.*** **By Dr. QUADRI, of Naples. Communicated in a Letter from Dr. SOMERVILLE to the President.**

Perhaps we ought to make some apology to our readers for dignifying with notice this memoir by Dr. Quadri; but we have been in some degree led to do this by the opportunity it offers us of noticing some interesting circumstances connected with Italian surgery.

Dr. Quadri commences by observing,

"Having maturely considered the nature of the gozzo; the uncertainty, to say the least, of its cure by the use of internal remedies, as well as the dangers incident to its extirpation by the knife, it occurred to me to try the effect of passing a seton through the substance of the enlarged gland, with the view of exciting suppuration, and also such a degree of irritation as might stimulate the absorbent vessels to remove a portion of it."

Seven cases are here related in which this practice was tried. In the first experiment, the circumference of the neck was reduced, under this treatment, from sixteen inches to thirteen inches and six lines. In the two succeeding, the reduction was not quite an inch, although in one of them the seton was enlarged eight times, and various acrid and corrosive substances introduced with it,—as oil of turpentine, red precipitate, black hellebore, &c. and inflammation and suppuration excited by these means. The next case was a soft bronchocele; but Dr. Quadri does not say much about the results of his practice in this instance. The two next furnish no conclusive results.

The little advantage derived from this treatment will, we think, deter others from pursuing the same course. Neither, from the history of these cases, should we be led to conclude, that the little reduction of parts which did take place was effected by *absorption* generally throughout the tumor, as the author asserts, but merely by the ulceration immediately produced by the seton. This practice, too, has been tried in England, and without success. The writer of this critique proposed to Dr. ASSALINI, the associate and quondam preceptor of Dr. Quadri, to try the effect of ligatures on the thyroid arteries; as, in the case in which this measure was resorted to by Sir WILLIAM BLIZARD, there were sufficient evidences of the probability of its success, had not some accidental circumstances occurred; and nothing in the observations of Dr. Quadri tended to show that it might not be successful, if performed on the principal vessels by an expert surgeon.

The internal remedies enumerated by Dr. Quadri are, burned sponge, the animal oil of Dippel, burned woollen cloth, and muriate of lime; but, notwithstanding he has, he says, maturely considered the nature of bronchocele and the uncertainty of the efficacy of those remedies, he is unable to determine whether they fail, from inducing disorder of the stomach, from the difficulty of duly preparing and administering them, or from some species of goitre being of a nature not subject to their influence. We think that very superficial observation might have removed those doubts.

From having seen numerous examples of bronchocele in many parts of Europe, a multitude of circumstances burst on our minds respecting the probable origin and nature of this extraordinary affection; but the limits of this Journal will not, on the present occasion, permit us to arrange our ideas on this subject: we shall, however, probably embrace some future opportunity to offer what, if it do not immediately elucidate the question, will point out those circumstances to future travellers that promise to afford the means of developing the causes of this disease.

Many of our readers, who have never quitted this happy island, may not be aware that the medical character on most parts of the continent does not rank higher than that of a master tailor or mechanic in this country. The army being the only occupation highly honoured, the grand tide of talents is directed to that profession. Medical men, with a few luminous exceptions, are not often men of deep erudition, and but rarely of good families; and, in order to recommend themselves, we have heard them publicly extolling their own merit. We have often been obliged to remind Sig. Assalini that many points of surgery he professed to have introduced here, were known amongst us long before he favoured us with a visit. Still Sig. Assalini is proud of the honour here conferred on him; and we have seen him wear as an order, suspended by a neat ribbon, a silver medal, conferred on him by the Society for the Encouragement of Arts, for his invention of some surgical instruments: this, he says, he deems far more valuable than those he received from Napoleon; for, he observed, it had never been conferred even on an Englishman: and he has had his portrait elegantly engraved, and this bauble occupies the most distinguished place in the picture. As the name of Mr. Quadri has not been known amongst us, it may be proper to observe, that he has lately arrived from Bologna to practise as an oculist at Naples, and is considered to be in partnership with Sig. Assalini.

We shall only relate one circumstance to show the state of surgical knowledge in Naples, and the clerical dominion to which it is there obliged to submit. On our arrival there, we were surprised to find that the use of the single ligature was unknown, as well as the mode of healing wounds by the first intention. At our instigation, various experiments were performed at the Veterinary College, to show the efficacy of the former measure. The professional men who attended these experiments were so satisfied with their results, that they obtained permission to try what they termed *the new method*, in two cases of popliteal aneurism: but these, unfortunately, had a fatal termination; on which occasion, his Reverence, who acted as guardian to the hospital, forbid the repetition of similar measures. We had, therefore, the artery carefully guarded from injury, in such cases, by surrounding linen, &c. and the wound healed from the bottom as heretofore. In justice, however, to Sig. PENZA, the operating surgeon, we must observe, that he referred the death of the patients to causes independent of the operation, and deeply regretted the arbitrary direction of the priest, and the degraded state of his profession in being obliged to yield to his decision.

***On Elephantiasis, as it appears in Hindostan.*** By JAMES ROBINSON, Esq. Superintendant of the Insane Hospital at Calcutta. Communicated by Dr. BABINGTON.

As our predecessors consigned to the department of this Journal appropriated chiefly to illustrations of the history of medicine, their selections of the descriptions given of this species of disease as it affected the Greeks and Arabs, and as it has lately been witnessed in Madeira and in England, we shall also insert this interesting complement to the history of Elephantiasis in our *Collectanea*, on some future occasion.

***Observations on Diseases of the Teeth.*** By THOMAS BELL, Esq. F.L.S. Communicated by Mr. TRAVERS.

This paper contains several useful remarks, adduced apparently from correct observation: we shall briefly state the most prominent. Mr. Bell first relates two cases to show that, contrary to the opinion of M. BLAINVILLE, and some other respectable physiologists, the crown of the tooth is possessed of vitality; in other words, that it is not a mere inorganic concretion. The first was an affection of one of the molares, attended with the most excruciating pain. The alveolar process and gums had undergone absorption, although there was not any external appearance of disease in the tooth. On sawing through the crown of the tooth transversely, after it was removed from the jaw, Mr. Bell found a cavity formed in the bony substance of the tooth, communicating with the natural cavity, and filled with pus. There was no discoloration nor appearance of caries in the surrounding part of the tooth. Mr. Bell thus accounts for this disease: "Here was a distinct case of abscess found in the bone of a tooth: the membrane had been inflamed, suppuration followed, and the pressure of the pus occasioned *absorption*." The other case was one in which a small portion of enamel was splintered off from the surface of an incision, by which the bone was exposed. Touching this point with any sharp instrument, occasioned pain of the most pungent kind, although there was not the least general tenderness in the tooth itself. The results of Dr. BLAKE's experiments, showing that the teeth became tinged with red on feeding young animals with madder, and that this colour afterwards disappeared on omitting the use of that substance, seem alone to prove the same fact in a satisfactory manner.

From the slight degree of vitality, however, possessed by the crown of the tooth, Mr. Bell infers the cause of the caries affecting it generally, on any portion of it becoming inflamed, or otherwise injured; a little morbid action being sufficient to destroy it, and that action becomes, as is the case in other parts, generally diffused when it occurs in any particular portion of the organ, from the part already affected irritating the surrounding structure. And, he further argues, the same principle will explain why "caries invariably commences at the external part of the bone, immediately under the enamel;" this part possessing the least degree of vitality, except the enamel itself. On the other hand, the *fang*, being more vascular, is not so readily affected with caries. Mr. Bell also shows, that the opinion vulgarly entertained, that a decayed tooth will produce disease in one in contact with it, is erroneous; and has arisen from the affection of both having a common origin, though the consequent disease has been manifested earlier in one tooth than in the other.

Mr. Bell adduces some interesting observations, too, on inflammation of the periosteum of teeth; arising, not from caries, but from more remote causes. Effusion of coagulable lymph between the fangs of the teeth and the alveolar processes, and the formation of abscesses, are the common results, if the disease be not efficaciously treated. Lancing the gums, leeches to those parts and to the integuments covering the jaws, vesicatories behind the ears, purgatives, and other antiphlogistic measures, are the curative means to be employed.

